# Effects of Elastic Tape Associated With Pulmonary Rehabilitation in Male Individuals With Chronic Obstructive Pulmonary Disease: Protocol for a 2-Arm, Assessor-Blinded Randomized Controlled Trial

**DOI:** 10.2196/75029

**Published:** 2026-02-27

**Authors:** Thiago Fernandes Pinto, Juliana de Melo Batista dos Santos, Estéfane Caroline Monteiro Reis, Cibele C Berto Marques da Silva, Fabiano Francisco de Lima, Regina Maria Carvalho-Pinto, Celso Ricardo Fernandes Carvalho

**Affiliations:** 1 Department of Physical Therapy, Medical School, University of São Paulo, Rua Cipotânea, 51, Cidade Universitária, São Paulo, 05360-160, Brazil, 55 1198415-3234; 2 Health Sciences Post-Graduation Program, Santo Amaro University - UNISA, São Paulo, Brazil; 3 Pulmonary Division, Heart Institute (InCor), Clinics Hospital, Medical School, University of São Paulo, São Paulo, Brazil

**Keywords:** chronic obstructive pulmonary disease, COPD, adhesive elastic tape, pulmonary rehabilitation, dyspnea, exercise capacity, physical capacity

## Abstract

**Background:**

Individuals with severe chronic obstructive pulmonary disease (COPD) may exhibit thoracoabdominal asynchrony, which reduces ventilatory efficiency. A novel intervention using elastic tape (ET) applied to the chest wall has been shown to acutely reduce thoracoabdominal asynchrony and dyspnea during exercise among individuals with COPD. We hypothesize that using ET in pulmonary rehabilitation (PR) may increase the benefits of PR in this population.

**Objective:**

This study aims to evaluate the additional effects of ET on exercise capacity, symptoms of anxiety and depression, health-related quality of life, and physical activity in daily life among male individuals with moderate to very severe COPD who are undergoing PR.

**Methods:**

This is a protocol for a randomized, controlled, 2-arm, parallel, blinded assessor clinical trial. Individuals will be followed for 8 weeks, twice a week, with PR sessions lasting approximately 1 hour. Health status (COPD Assessment Test), health-related quality of life (Chronic Respiratory Questionnaire), and psychological distress (Hospital Anxiety and Depression Scale) will be assessed before and after the intervention. Then, exercise capacity will be assessed via the incremental shuttle walking test and endurance shuttle walking test, and participants will use a triaxial accelerometer (ActiGraph) for 7 days to assess physical activity in daily life. Subsequently, individuals will be randomized into ET or sham groups; both groups will complete a PR program (2 times per week for 8 weeks). The ET group will receive applications of ET, whereas the sham group will receive a nonelastic tape. Data will be presented as means and SDs or medians and IQRs. Intergroup comparisons will be performed using a 2-way ANOVA, followed by the Bonferroni post hoc correction test, or the Kruskal-Wallis test, followed by the Dunn post hoc test. The threshold for statistical significance will be set at 5%.

**Results:**

The clinical trial registration was approved in June 2023. Recruitment and data collection for the trial are ongoing; as of November 2025, a total of 10 individuals have been recruited, and the results are expected to be available by the end of November 2026.

**Conclusions:**

We hypothesize that the use of ET can enhance the benefits of PR in individuals with moderate to very severe COPD and increase exercise capacity and quality of life, as well as reduce symptoms of anxiety and depression.

## Introduction

Chronic obstructive pulmonary disease (COPD) is a preventable and treatable disease characterized by persistent and progressive airflow limitations associated with a chronic inflammatory response of the airways [1]. In the PLATINO (Projeto Latino-Americano de Investigação em Obstrução Pulmonar) study conducted in 2005, the prevalence of COPD was 15.8% (95% CI 13.5%‐18.1%) using the fixed ratio definition. COPD showed a positive association with age and smoking and an inverse association with BMI; thus, we understand that most individuals with COPD in the Brazilian population are not obese [2]. These findings were consistent with a study published in 2019 [3], which showed that individuals with moderate to very severe COPD had a slight reduction in BMI. The same 2019 study [3] showed that there was a similar prevalence for both sexes. Individuals with COPD experience destruction of the small airways and decreased elastic recoil, leading to air trapping at rest due to the loss of the intrinsic tendency of the lungs to deflate. Movements of the thoracic and abdominal compartments may be impaired and may become worse with exertion [4]. Therefore, the rib cage and abdomen may move uncoordinately; this phenomenon is called thoracoabdominal asynchrony (TAA) [5].

TAA occurs in individuals with severe airway obstruction, leading to a mechanical disadvantage, increased breathing work, dyspnea symptoms, limited exercise, and reduced physical activity in daily life (PADL) [6,7]. To our knowledge, few studies have found interventions to minimize TAA. Yamaguti et al [8] found that a diaphragmatic breathing retraining program could reduce TAA among individuals with COPD. Recently, Pinto et al [9] accomplished a novel intervention using elastic tape (ET) in the chest wall and demonstrated that ET reduces TAA, tidal volume, minute volume, and dyspnea during isoload exercise among individuals with severe to very severe COPD. Individuals also demonstrated improvements in maximal oxygen consumption during cardiopulmonary exercise testing and a reduction in sedentary time during daily life. More recently, Santos et al [10] used ET for 3 weeks in patients with moderate to very severe COPD and reported reduced dyspnea symptoms as well as improved health status and health-related quality of life (HRQoL). Although these studies used ET, neither did so in conjunction with pulmonary rehabilitation (PR), and both studies had an intervention exposure duration of a maximum of 21 days. Furthermore, there is no evidence of ET being used in PR as an adjuvant strategy.

PR is an effective and successful treatment for individuals with persistent symptoms and/or excessive use of health care resources [11]. The European Respiratory Society and the American Thoracic Society define PR as a comprehensive intervention based on a thorough patient assessment followed by patient-tailored therapies, which include but are not limited to exercise training, education, and behavior changes designed to improve the physical and psychological conditions of people with chronic respiratory disease and to promote long-term adherence to health-enhancing behaviors [12,13]. The core of PR is exercise training, which directly addresses deconditioning and its association with decreases in symptoms such as dyspnea and fatigue, which are the primary reasons for referral. Exercise training programs also have additional positive effects on comorbidities, HRQoL, and general health [13]. However, the additional effects of ET associated with PR have not been studied.

Although previous studies have investigated the use of ET in isolation [9,10], and considering that ET has been shown to reduce TAA [9], our hypothesis is that the application of ET during a PR program may confer additional positive effects in individuals with greater disease severity regarding exercise capacity, symptoms of anxiety and depression, and HRQoL in individuals with moderate to very severe COPD undergoing PR.

## Methods

### Study Design and Participants

This is a protocol for a randomized, controlled, 2-arm, parallel, assessor-blinded clinical trial. Individuals with COPD will be screened and recruited at the pulmonology outpatient clinic of a tertiary hospital. The study protocol was developed in accordance with the SPIRIT (Standard Protocol Items: Recommendations for Interventional Trials) checklist guidelines.

### Analysis of the Population

The inclusion criteria will be as follows: diagnosis of moderate to very severe COPD [1], clinical stability (ie, no exacerbations for at least 30 days), male sex, nonobese status (BMI ≤29.9 kg/m^2^), absence of musculoskeletal limitations, and no participation in another research protocol or a PR program in the past 6 months. The exclusion criteria will be decompensated cognitive, neurological, and/or cardiological dysfunction and receiving home oxygen therapy. An intention-to-treat analysis will be performed using the patient’s most recent assessment in case of study dropout or absence of data and will be treated by imputing the last observation carried forward [14].

### Ethical Considerations

Patients will be recruited from the pulmonology outpatient clinic of the Hospital das Clínicas de São Paulo, São Paulo, Brazil, and will be included in the study after signing the informed consent form. This study will be performed in accordance with the Declaration of Helsinki. The study was registered at ClinicalTrials.gov (NCT05939999), and the Hospital Research Ethics Committee approved the study (55617321.2.0000.0068). All participants will be informed about the possibility of withdrawing at any time without incurring any loss of treatment at the institution. Participants will receive an identification code according to the order of inclusion in the study. The data will be deidentified by excluding the main identifying elements such as name, address, date of birth, phone number, email, medical record number, institutional identification number, and social security number. In addition, participants will be informed about the voluntary nature of their participation; this means that there will be no compensation of any kind for participation.

### Randomization and Allocation Concealment

The randomization will be generated using a computer (Sealed Envelope [15]). An investigator who is not involved in patient recruitment, assessment, or intervention will perform group allocation. Group allocation will be concealed via consecutively numbered, sealed, opaque envelopes. After randomization, both groups (ET vs sham) will not be informed of their allocation, and the same instructions on perception of tape use will be given to both groups.

### Adverse Events

The individual’s skin condition will be assessed at every session during the PR program to immediately identify any allergic reactions to the tape in both groups. If any skin changes are identified, such as redness or itching, the tape will be removed immediately and will not be reapplied until the condition has been completely resolved. If the condition does not resolve within 72 hours, even after removing the tape, the individual will be referred for evaluation by a dermatologist and will be removed from the study.

### Experimental Design

Clinical and anthropometric data will be collected during the participant interview. Participants will complete questionnaires to assess health status (COPD Assessment Test [CAT]), HRQoL (Chronic Respiratory Questionnaire [CRQ]), and anxiety and depression symptoms (Hospital Anxiety and Depression Scale [HADS]). Then, a complete pulmonary function test (body plethysmography), the incremental shuttle walking test (ISWT), and the endurance shuttle walking test (ESWT) will be carried out. Finally, individuals will use a triaxial accelerometer (ActiGraph GT9X) for 7 days to assess their PADL. Lastly, individuals will be randomly assigned to 1 of 2 groups: the ET group or the sham group.

**Figure 1.**
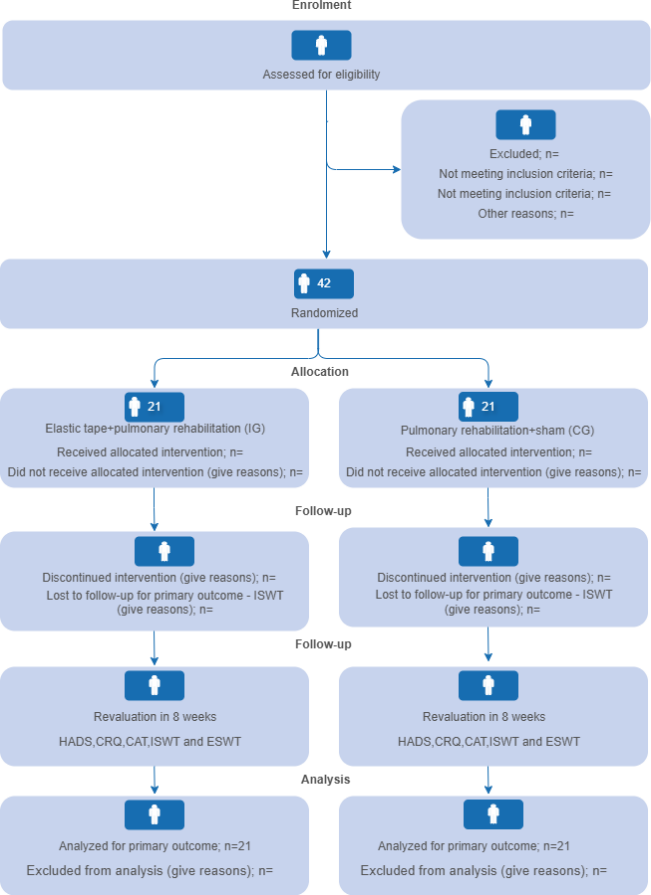
CONSORT (Consolidated Standards of Reporting Trials) 2025 flow diagram showing the enrollment, allocation, follow-up, and analysis phases of the randomized controlled trial. Participants were assessed for eligibility, randomized into 2 groups—elastic tape+pulmonary rehabilitation (intervention group [IG]) or pulmonary rehabilitation alone (control group [CG])—and reevaluated after 8 weeks. The number of patients shown in the figure is the planned number based on the sample size. Outcome assessments included the Hospital Anxiety and Depression Scale (HADS), Chronic Respiratory Questionnaire (CRQ), and COPD Assessment Test (CAT). ESWT: endurance shuttle walking test; ISWT: incremental shuttle walking test.

**Figure 2.**
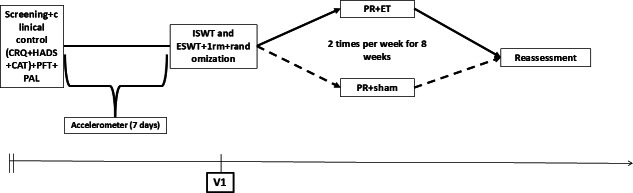
Schematic representation of the study design. Assessments were performed at baseline and after 8 weeks of intervention. 1RM: 1 repetition maximum test; CAT: COPD Assessment Test; CRQ: Chronic Respiratory Questionnaire; ET: elastic tape; ESWT: endurance shuttle walking test; HADS: Hospital Anxiety and Depression Scale; ISWT: incremental shuttle walking test; PAL: physical activity level; PR: pulmonary rehabilitation; PFT: pulmonary function test; V: visit.

At baseline and postintervention, assessments will be performed by a researcher who is not involved in individual recruitment or intervention. After inclusion, individuals will begin an 8-week PR program twice a week (Figure 1). Individuals in the ET group will have the ET placed before the beginning of the PR program, and the ET will be replaced every 7 days throughout the 8-week period. The sham group will receive a nonelastic tape of the same color and size at the same position in the chest wall at the beginning of the PR program, and the tape will be replaced every 7 days, similar to the ET group. At the end of the PR program, participants will repeat the same assessments, except for the ISWT. The ESWT will be performed at the same level established by the ISWT during the initial evaluation (Figure 2).

### Intervention

#### ET Overview

**Figure 3.**
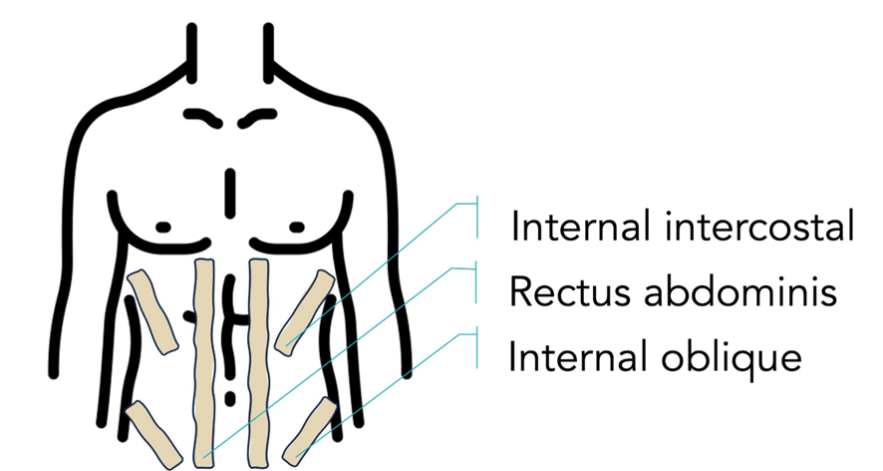
Illustration of the elastic tape placement protocol. Tape was applied to key expiratory muscle groups—rectus abdominis, internal oblique, and intercostal muscles—using standardized positioning and tension to ensure reproducibility and comfort during the pulmonary rehabilitation sessions.

The ET (Kinesio Brand Tape, Tex Gold), which is made of cotton and elastic material and is skin-colored and waterproof, will be applied by the same trained professional throughout the study to avoid intervention fidelity bias, with the participant in a relaxed, supine position. The individual will lie down on a stretcher so that their torso is extended, thus facilitating the application of the ET [9]. The ET will be positioned at the origins and insertions of the following muscle groups: rectus abdominis, internal oblique, and internal intercostal (Figure 3). These muscle groups were chosen because they are the main muscles involved in forced expiration during exercise, and the tape will always be placed with the maximum possible tension, which is 200% of its original size. The tape will be placed at the beginning of the session and replaced every 7 days. If at least 2 cm of tape is detached, the ET will be replaced regardless of the length of time it has been in place.

#### Sham Group

The sham group will receive a nonelastic tape (3M Micropore) placed using an identical procedure to the ET and replaced every 7 days. If at least 2 cm of tape is detached, the tape will be replaced regardless of the length of time it has been in place.

#### PR Program

On the first day, at the start of PR, individuals will be briefed on the benefits of PR, such as reduced dyspnea, additional positive effects on comorbidities, improved HRQoL and overall health, and increased exercise capacity [13]. Additionally, a WhatsApp contact will be provided on the same day for ongoing communication regarding health-related issues. Both groups will undergo an 8-week PR program, including 1-hour sessions twice a week, supervised by a physiotherapist with experience in PR, carried out in the hospital’s outpatient clinic. The program will be individualized and divided into 30 minutes of aerobic training on an ergometric treadmill (Technogym Jog 500) and 30 minutes of resistance training using free weights (dumbbells and barbells) for the upper limbs and gym machines for the lower limbs (LLs). Aerobic training will be performed with a heart rate (HR) corresponding to 50% to 80% of the maximum HR according to the formula proposed by Karvonen et al (HR_training_ =HR_rest_+ [HR_max_–HR_rest_]×% intensity), where HR_training_ is training heart rate, HR_rest_ is resting heart rate, HR_max_ is maximum heart rate, and % intensity is the percentage of training intensity. The intensity will be increased weekly by 5% of the maximum HR until reaching 80% of the maximum HR. Furthermore, this progression of treadmill load will also be guided by the scale of subjective perception of exertion (modified Borg scale) [16]. The training will be carried out in such a way that during each session, the participant must remain on the Borg scale between scores 4 and 6 [17]. The increase in intensity by 5% of the maximum HR will be adjusted after 2 sessions and/or if the score on the Borg scale is less than 4. If the individual reports fatigue greater than 6 on the modified Borg scale, the intensity will be reduced and, in the next session, the increase in intensity will be tried again. The individual may interrupt the activity if they feel symptoms or intense respiratory discomfort, resuming it after improvement. HR, peripheral oxygen saturation (SpO_2_), and the subjective perception of exertion will be monitored during all exercise. Oxygen supplementation will be provided as needed to maintain SpO_2_ of 90% or greater [17].

Resistance training will consist of exercises for the upper limbs through lifting weights with dumbbells and for the LLs via weight machines. Large muscle groups will be trained, including the biceps, deltoids, quadriceps, and hamstrings. Training will begin with 3 sets of 8 repetitions of each exercise at a workload of 50% of the one-repetition maximum [18]. The number of repetitions will subsequently increase to 3 sets of 10 and, later, 12 repetitions. Load progression will be carried out when the individual tolerates performing more than 12 repetitions for each set without compensatory movements and/or symptoms of intolerance. The workload will be increased according to individual tolerance.

Resistance training for the upper limbs will include shoulder abduction and elbow flexion. Resistance training for the LLs will include knee extension and hip extension using “leg press” equipment.

No written material will be provided to either group. Transportation expenses will be subsidized for all individuals to reduce absenteeism and increase feasibility and adherence to the protocol. This financial resource will be funded for this research project. For analysis of the results, individuals must complete at least 80% (13 sessions) of the program, which will be monitored using the PR monitoring form, where data on HR, SpO_2_, the Borg scale, speed, and treadmill incline will be tabulated every 5 minutes, from the beginning to the end of the training session for all sessions.

The acceptability of ET use will be monitored throughout the session based on a visual inspection performed by the physiotherapist at the beginning of the session and during ET replacement every 7 days to immediately identify any skin changes that the individual may experience due to continued ET use.

To ensure or increase adherence to the 8-week PR program, the importance and potential benefits of PR, as established in the literature, will be explained to each participant individually, regardless of the group they are allocated to after randomization. Throughout the PR program, individuals will have a WhatsApp contact available to clarify and assist with any questions regarding their COPD-related health status. In cases of clinical worsening, the individual will be taken to the hospital emergency room for immediate medical care.

### Assessment Tools

#### Primary Outcome Measure: Functional Exercise Capacity

The ISWT will be performed to assess functional exercise capacity. In this test, individuals will be instructed to walk in a 10 m corridor delimited by cones (with a 9 m distance between them), and the speed will be determined by a beep recorded on the sound box, which increases the speed by 0.17 m/s every minute, with a total of up to 15 stages. They will also be instructed to walk at a speed that allows them to reach the cones when the beep sounds, and at the end of each minute, an additional signal will be given to alert them to the increase in speed [19]. The test will end when the participant is more than 0.5 m away from the cone, reports chest pain, reports severe dyspnea, reports fatigue, reports exhaustion, or requests to stop. The distance walked at the end of the test will be registered for subsequent analyses [20]. Before and after the exercise tests, SpO_2_ and HR will be assessed via a portable pulse oximeter positioned on the individual’s dominant hand (WristOx2 3150; Nonin). Blood pressure (BP) will be assessed via a stethoscope (Littman) and a sphygmomanometer calibrated on the dominant arm (Aneroid Premium - G-Tech).

The criteria for stopping the test will include diastolic BP (DBP) above 140 mm Hg, sustained elevation of systolic BP (SBP) above 240 mm Hg, motor incoordination, feelings of imbalance, mental confusion, clinical manifestations of respiratory distress that worsen with increasing load, dyspnea disproportionate to the intensity of the effort, progressive claudication of the LL, and limb exhaustion [12]. Individuals will be instructed not to consume beverages containing caffeine for at least 12 hours before the test and not to perform intense exercise 24 hours before.

The ESWT will be performed 40 minutes after the end of the ISWT, using the same structure; that is, the volunteers will be instructed to walk in a 10m corridor limited by cones (with a distance of 9 m between them), and the speed will be determined by a sound signal recorded by the cell phone. In this case, the speed will be determined using 85% of the maximum speed reached in the ISWT and will be constant throughout the test [12]. Individuals will be monitored before and after the test, and the interruption criteria will be the same as those used in the ISWT procedure.

#### Secondary Outcome Measures: Health-Related Quality of Life (Chronic Respiratory Questionnaire – CRQ)

This questionnaire is already validated and translated into Portuguese [21]. It consists of 20 questions divided into 4 domains: fatigue (4 questions), dyspnea (5 questions), emotional function (7 questions), and self-control (4 questions). The responses will be assessed via a Likert scale, with 7 alternatives ranging from “never” to “always,” whereas the dyspnea domain includes an individualized assessment in which the patient chooses, from a list of 26 daily activities, the 5 activities that cause the most dyspnea and then rates each one again via a Likert scale with 7 alternatives ranging from “unbearable shortness of breath” to “no shortness of breath.” The results will be expressed as the mean score of each domain and the total score, with higher scores indicating better HRQoL.

### Anxiety and Depression Symptoms

The HADS will be used to assess anxiety and depression symptoms. The questionnaire was developed to identify symptoms of anxiety and depressive mood and has been translated, validated, and published in Portuguese by Botega et al [22]. It consists of 14 multiple-choice questions divided into 2 subscales, anxiety (HADS-A) and depression (HADS-D) symptoms, with 7 questions each. The domains are categorized by the severity of the stress present: none, 0‐7; probable, 8‐10; and present, ≥11 [23,24].

### Health Status

Health status will be assessed by the CAT, a specific questionnaire for COPD that assesses the impact of the disease’s symptoms [1]. It consists of 8 items related to health conditions and has been applied and validated in several languages, including Portuguese [25].

### Sample Characterization

#### Initial Assessment

Individuals will undergo an initial interview to provide information on several aspects: identification and contact data, medical history (eg, depression, osteoarthritis or arthrosis, osteoporosis, diabetes mellitus, and arterial hypertension), and current medications.

#### Lung Function

Pulmonary function parameters will be obtained from the individual’s medical records within the previous 6 months. The interpretation will be performed according to the standards of the American Thoracic Society and European Respiratory Society [26] and normal values relative to the Brazilian population [27].

#### Physical Activity in Daily Life

The PADL will be quantified using a triaxial accelerometer (GT9X; ActiGraph) for sample characterization. The equipment will be placed on the individual’s waist, and the individual will be advised to wear it for 7 days and only remove it to sleep and shower. They will be instructed to maintain their lifestyle habits. Seven full days of monitoring will be considered to evaluate the PADL: 5 weekdays and 2 weekend days. Zero counting time (considered the period in which the participant did not use the accelerometer) or days with less than 8 hours of device use will be excluded [28]. The results will be expressed as the time of sedentary behavior, steps per day, and time spent in moderate to vigorous physical activity.

#### Statistical Analysis

Data normality will be assessed using the Shapiro-Wilk test, and homoscedasticity will be assessed using the Levene test. Continuous variables will be presented as mean (95% CI) or as medians and IQRs, according to their distribution. Intergroup comparisons will be performed using 2-way ANOVA for repeated measures with Bonferroni post hoc correction or the Kruskal-Wallis test with Dunn test when assumptions are violated. The proportion of individuals not reaching the minimal clinically important difference will be compared using the chi-square test. Within-group effect sizes (Cohen *d*) will be classified as small (0.2), moderate (0.5), or large (0.8). The Spearman correlation coefficient will be used to analyze the associations between changes in ESWT time and changes in CAT, HADS-A, HADS-D, and CRQ scores. Analyses will be conducted according to the intention-to-treat principle, using GraphPad Prism version 5.0, with significance set at *P*<.05.

#### Sample Calculation

The sample size calculation was based on the primary outcome, based on a previous study showing a mean difference of 65 (SD 65) seconds between groups in the ESWT [29]. Thus, considering an alpha of .05 and a beta of .80, the estimated sample size will be 17 individuals per group. To account for potential losses and to allow for data imputation under the intention-to-treat principle, the sample size was increased by 20% in each group. Therefore, the final estimated sample size was 21 participants per group.

## Results

The clinical trial registration was approved in June 2023. Recruitment and data collection for the trial are ongoing; as of November 2025, a total of 10 individuals have been recruited, and the results are expected by November 2026.

## Discussion

### Anticipated Findings

The use of ET during rest periods does not alter lung volume; however, during exercise, individuals with COPD under the same workload (isoload) have shown a notable decrease in tidal volume, minute volume, dyspnea, and TAA. Furthermore, individuals using ET have shown a significant increase in oxygen consumption, workload, and maximal exercise duration compared with those not using ET. Additionally, ET increased the amount of time spent in moderate and vigorous physical activity, along with a marked reduction in sedentary time. As the measurements were randomly performed with or without ET, these data strongly suggest that ET attenuated the ventilatory demand required for exercise performance across individuals [9].

Recently, Santos et al [10] demonstrated that ET use for 21 consecutive days does not alter PADL but reduces dyspnea and improves health status and HRQoL in individuals with moderate to very severe COPD in the short term. Therefore, this new, low-cost intervention also improves COPD symptoms during its continuous use. The use of ET may enhance the already-known effects of the PR program, such as increased exercise capacity and improved quality of life among individuals with COPD.

One of the strengths of the proposed study is the use of ET throughout the PR period (16 sessions over 2 months), which may improve the HRQoL of individuals with COPD. Furthermore, this is a randomized, controlled, 2-arm, and blinded study, making it the first study to evaluate the potential effects of ET throughout a PR program. Among the study’s limitations is the inclusion of only nonobese male individuals, which followed the same design as previously published studies [9,10]; however, our goal was to minimize bias, so we did not include obese individuals due to the protective effect of obesity on the respiratory mechanics of patients with COPD [30]. Therefore, future studies should include obese individuals and both male and female individuals. The single-center design and the inclusion of individuals who do not use oxygen therapy will make it difficult to generalize the interpretation of the data at the end of the study. The secondary outcomes, such as HRQoL, anxiety, and depression, were classified as exploratory. Therefore, these outcomes were used for a more comprehensive understanding of the intervention’s effects; however, the statistical analysis for these aspects may not have sufficient statistical power to yield definitive conclusions, and the results regarding secondary outcomes should be interpreted with due caution. For this reason, the results for these outcomes should be interpreted as preliminary insights, suggesting the need for future investigations into these outcomes, especially in female participants, as COPD affects both sexes similarly.

### Conclusions

We hypothesize that the use of ET can enhance the benefits of PR in individuals with moderate to very severe COPD and increase exercise capacity and quality of life, as well as reduce symptoms of anxiety and depression.

## Abbreviations

BP: blood pressure
COPD: chronic obstructive pulmonary disease
CRQ: Chronic Respiratory Questionnaire
ESWT: endurance shuttle walking test
ET: elastic tape
HADS: Hospital Anxiety and Depression Scale
HR: heart rate
HRQoL: health-related quality of life
ISWT: incremental shuttle walking test
LL: lower limb
PADL: physical activity in daily life
PLATINO: Projeto Latino-Americano de Investigação em Obstrução Pulmonar
PR: pulmonary rehabilitation
SPIRIT: Standard Protocol Items: Recommendations for Interventional Trials
SpO_2_: peripheral oxygen saturation
TAA: thoracoabdominal asynchrony
